# Iatrogenic botulinum Toxin Type A poisoning with persistent unilateral vocal cord paralysis: a case report and clinical implications

**DOI:** 10.3389/fphar.2026.1786491

**Published:** 2026-05-11

**Authors:** Shizhao Xiang, Yu Du

**Affiliations:** 1 Department of Critical Care Medicine, West China School of Public Health, West China Fourth Hospital, Sichuan University, Chengdu, Sichuan, China; 2 Health Emergency Management Research Center, China-PUMC C.C.Chen Institute of Health, Sichuan Univetsity, Chengdu, Sichuan, China

**Keywords:** botulinum toxin, botulinum Toxin Type A, botulism, iatrogenic injection, vocal cord paralysis

## Abstract

With the continuous advancement of medical aesthetic technologies, botulinum toxin injection has emerged as a prevalent method for wrinkle removal and facial enhancement. However, improper administration can result in botulism incidents. This article presents a case of poisoning caused by the facial injection of Botulinum Toxin Type A. The patient was successfully treated through comprehensive measures, including the administration of Botulinum Antitoxin Type A, neurotrophic therapy, and functional rehabilitation; however, unilateral vocal cord paralysis persisted. This case underscores the potential for severe complications, such as vocal cord paralysis, arising from improper injections of Botulinum Toxin Type A. Additionally, this article reviews the existing literature concerning vocal cord paralysis.

## 1 Introduction

**FIGURE 1:**
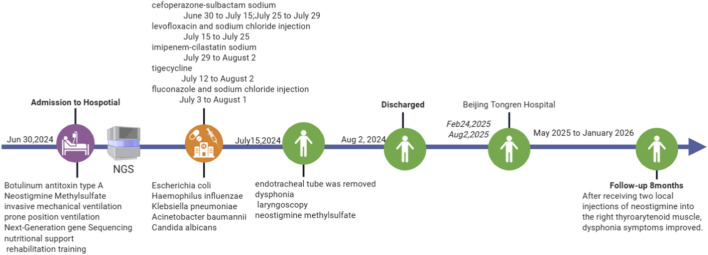
Course of treatment.

In daily life, approximately 80% of botulism cases arise from improper ingestion, primarily linked to environmental hygiene in food production, inadequate storage, and poor dietary habits (Freeman et al., 2020). Botulinum toxin, a potent biological neurotoxin widely utilized in the field of medical aesthetics, can lead to botulism if misused (Kılboz B et al., 2023; Fan K et al., 2016). Clinically, patients with botulism typically exhibit symptoms such as dizziness, blurred vision, diplopia, difficulty closing the eyes, dysarthria, dysphagia, choking while drinking water, and limb weakness. Severe cases may progress to respiratory muscle paralysis (Goerttler T et al., 2024), with a mortality rate as high as 5%–10% (Guo J et al., 2016). Vocal cord paralysis following the injection of Botulinum Toxin Type A is rarely reported. This article presents a case of unilateral vocal cord paralysis that persisted after successful treatment of poisoning caused by the facial injection of Botulinum Toxin Type A in our hospital and reviews the relevant literature on vocal cord paralysis (Figure 1). The objective is to raise awareness among individuals seeking aesthetic treatments and relevant practitioners regarding the potential risks associated with the injection of Botulinum Toxin Type A.

## 2 Case presentation

**FIGURE 2:**
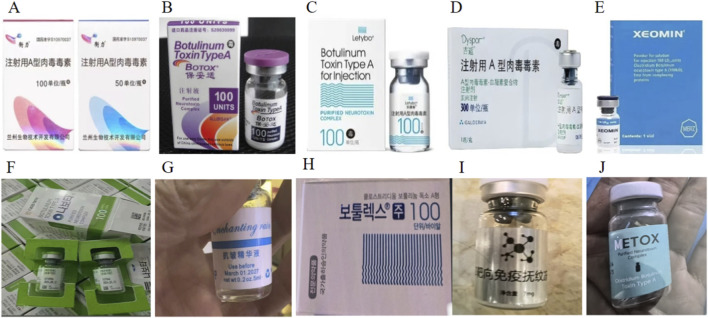
Legal brands of Botulinum Toxin Type A **(A–E)**. Hengli-China **(A).** Botox-USA **(B)**. Letybo-South Korea **(C)**. Dysport-UK **(D)**. Xeomin- Germany **(E)**. Illegal brands of Botulinum Toxin Type A **(F–J)**.

A 20-year-old female patient was admitted due to dysphagia and dyspnea that had persisted for 7 days. Seven days prior (30 June2024), she had received her first bilateral masseter injection of an illegal Botulinum Toxin Type A (Figures 2F-J) product (BOTULINUM TOXIN TYPE A 100 UNITS/1 VIAL, Batch No. X21090A, South Korea), totaling 200 units (100 units per side) at a local aesthetic institution (Figure 2F). The injection process proceeded smoothly without immediate discomfort.

However,1 day post-injection,she gradually developed dyspnea, dizziness upon standing, general weakness, bilateral ptosis, difficulty swallowing and chewing,and experienced choking episodes while drinking water. Notably, there were no accompanying symptoms such as fever, headache, nausea, vomiting, abdominal pain, or diarrhea. She self-administered ‘Mecobalamin Tablets’ without any improvement, and her symptoms progressively worsened. Five days post-injection, she visited ‘Beijing 307 Hospital,’ where a preliminary diagnosis of ‘botulism’ was made. Due to the unavailability of Botulinum Antitoxin Type A serum at that facility, only symptomatic treatment was provided. As her swallowing and breathing difficulties further deteriorated 6 days post-injection, she was admitted to ‘Beijing Aviation General Hospital.’ Botulism remained a consideration. However, once again, due to the lack of antitoxin serum, only anti-infection and neurotrophic treatments were administered. Given the poor response to treatment and upon learning that our hospital had the antitoxin serum, a transfer to our facility was recommended. The patient’s condition was critical, with a high risk of airway compromise, exhibiting peripheral oxygen saturation fluctuating between 88% and 92% on mask oxygen. Consequently, the transferring hospital performed protective endotracheal intubation prior to her transfer to our hospital.

**FIGURE 3:**
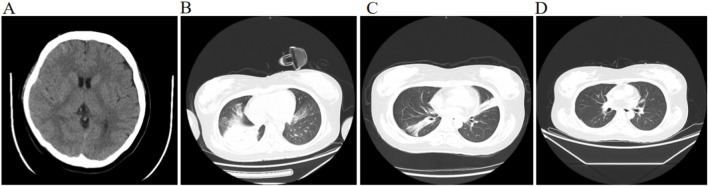
Head and Chest CT scan during hospitalization **(A–D)**. Head CT upon admission **(A)**. Chest CT upon admission **(B)**. Chest CT 2 weeks post-admission **(C)**. Chest CT 2 weeks post-discharge **(D)**.

On initial physical examination, the patient’s temperature was 36.8 °C, respiratory rate was 23 breaths per minute, heart rate was 115 beats per minute, and blood pressure was 122/78 mmHg (1 mmHg ≈0.133 kPa), with an oxygen saturation (SpO_2_) of 100% on 40% fractional inspired oxygen (FiO_2_). The patient was conscious and endotracheally intubated in SPONT mode, exhibiting an acutely ill appearance. No rash, petechiae, or ecchymosis were observed on the body. There was bilateral ptosis with difficulty in opening the eyes. The pupils were isocoric and round, approximately 3 mm in diameter, with a sensitive light reflex and no nystagmus. The neck was supple, though the patient experienced difficulty lifting the head. Moist rales were audible in both lungs. The cardiovascular examination revealed a regular rate and rhythm with normal heart sounds. The abdomen was soft, with no evidence of hepatomegaly or splenomegaly. Muscle strength was graded at 4 for the upper limbs and 3 for the lower limbs, with normal muscle tone and negative pathological signs. The initial head CT scan showed no abnormalities (Figure 3A), while the chest CT scan indicated inflammatory exudation and consolidation in the lower lobes of both lungs (Figure 3B). A blood test for botulinum toxin detected a positive result for Botulinum Toxin Type A (using ELISA and FICA methods, Test No. 20240701–13, Chengdu Borui Medical Laboratory, Shu ICP No. 2023022980).

**FIGURE 4:**
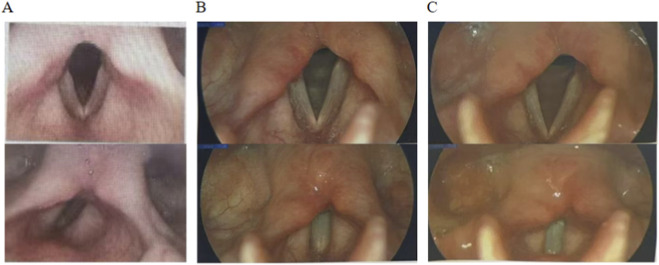
Stroboscopic evaluation of vocal fold vibratory characteristics **(A–C)**. 2 weeks upon admission **(A)**. 6 months post-discharge **(B)**. 1 year post-discharge **(C)**.

Upon admission, invasive mechanical ventilation was initiated (spontaneous breathing mode, with an inspired oxygen concentration of 40%). Botulinum antitoxin type A (specification: 10,000 IU/vial, National Drug Approval Number S10820152, Lanzhou Institute of Biological Products Co., Ltd.) was administered via intramuscular injection into the deltoid muscle of the upper arm at a dosesage of 10,000 IU every 12 h for a total of 7 days, along with Neostigmine Methylsulfate (specification: 0.5 mg/1 mL per vial, National Drug Approval Number H41022269, Hunan Runhong Pharmaceutical Co.,Ltd.) was given via intramuscular injection into the gluteal muscle at a dose of 0.5 mg every 12 h for a total of 10 days. Due to chest imaging indicating severe pneumonia, the patient underwent prone position ventilation and multiple bronchoscopic bronchoalveolar lavage (BAL). BAL fluid culture results were positive for *Escherichia coli*, *Haemophilus* influenzae, *Klebsiella pneumoniae*, *Acinetobacter* baumannii, and *Candida* albicans. Based on antimicrobial susceptibility testing, appropriate intravenous antibiotic therapy was administered as follows: cefoperazone-sulbactam sodium 3 g every 8 h from June 30 to July 15, and from July 25 to July 29; levofloxacin and sodium chloride injection 0.2 g every 12 h from July 15 to July 25; imipenem-cilastatin sodium 2 g every 8 h from July 29 to August 2; tigecycline 100 mg every 12 h from July 12 to August 2; and fluconazole and sodium chloride injection 0.2 g once daily from July 3 to August 1. Concurrently, nutritional support was strengthened, and passive rehabilitation training for limb muscle strength was performed at the bedside. After 2 weeks of treatment, follow-up chest CT showed significant improvement in the pulmonary infection (Figure 3C). The patient was successfully weaned from mechanical ventilation and the endotracheal tube was removed. On the day of extubation, the patient self-reported dysphonia and a low, weak voice. Bedside fiberoptic bronchoscopy revealed no airway abnormalities. However, specialized laryngoscopy indicated restricted mobility of the right vocal cord (Figure 4A), with no structural abnormalities of the vocal cords observed, suggesting right vocal cord paralysis. The patient received intramuscular gluteal injections of neostigmine methylsulfate (0.5 mg every 12 h for 5 days), which resulted in slight improvement of the vocal cord paralysis. The patient’s poisoning symptoms achieved clinical cure, and she was discharged on 2 August 2024. A follow-up chest CT performed 2 weeks after discharge demonstrated complete resolution of the pulmonary infection foci (Figure 3D). Six months post-discharge, with no significant improvement in phonation difficulty. She sought specialized care at Beijing Tongren Hospital, Capital Medical University. Examination confirmed the continued limited mobility of the right vocal cord (Figure 4B). One year after discharge, she returned to Beijing Tongren Hospital once again, reporting slight improvement compared to 6 months prior (Figure 4C). Laryngeal electromyography (LEMG) and evoked potential studies were conducted, revealing electrophysiological abnormalities in the right thyroarytenoid and posterior cricoarytenoid muscles (Tables 1, 2). Follow-up observation was recommended to allow for potential spontaneous recovery. During the subsequent 8-month follow-up period (from May 2025 to January 2026), the patient received two local injections of neostigmine into the right thyroarytenoid muscle at a specialized hospital. The dysphonia symptoms improved 3 days after each injection.

**TABLE 1:** Laryngeal electromyography (LEMG) findings.

Laryngeal muscle	Spontaneous activity & motor unit potentials			Motor unit recruitment	
	Type	Amplitude (uV)	Duration (ms)	Recruitment pattern	Max amplitude (uV)
Thyroarytenoid (left)	Normal	137	3.8	Interference	813
Thyroarytenoid (right)	Abnormal	121	4.5	Interference	413
Posterior cricoarytenoid (left)	Normal	256	4.5	Interference	1,206
Posterior cricoarytenoid (right)	Abnormal	246	5.4	Interference	932
Cricothyroid (left)	Normal	115	4.0	Interference	835
Cricothyroid (right)	Normal	119	4.1	Interference	797

**TABLE 2:** Laryngeal evoked potential analysis.

Laryngeal nerve	Laryngeal muscle	Latency (ms)	Duration (ms)	Amplitude (mV)
Recurrent laryngeal nerve (left)	Thyroarytenoid (left)	1.8	4.5	6.8
Recurrent laryngeal nerve (right)	Thyroarytenoid (right)	1.8	6.9	6.6
Recurrent laryngeal nerve (left)	Posterior cricoarytenoid (left)	1.8	4.8	9.5
Recurrent laryngeal nerve (right)	Posterior cricoarytenoid (right)	1.8	6.7	4.2
Superior laryngeal nerve (left)	Cricothyroid (left)	1.8	5.3	6.2
Superior laryngeal nerve (right)	Cricothyroid (right)	1.7	4.8	5.8

## 3 Discussion

This article reports the successful management of a case of injection-induced botulism. However,the patient developed residual focal laryngeal dysfunction (unilateral vocal cord paralysis) after the toxic and infectious symptoms were controlled, suggesting a lack of adequate clinical understanding of the toxicological characteristics, pathogenesis,and potential risks of iatrogenic botulism. Drawing on the literature, this article systematically elaborates on the toxicological basis of botulism,the unique features of iatrogenic poisoning,the pathological mechanisms of vocal cord paralysis,and clinical diagnostic and treatment strategies, aiming to provide a reference for the early recognition and standardized management of this disease.

Over the past two centuries, botulism has evolved from a foodborne catastrophe characterized by high mortality into a unique disease that poses both a public health threat and holds medical application value. Botulism is a severe neurological disorder caused by the botulinum neurotoxin (BoNT) produced by *C. botulinum*. This bacterium is a Gram-positive, obligate anaerobic,spore-forming rod widely distributed in global soil, marine sediments,and on the surfaces of vegetables, fruits, and seafood. Besides *Clostridium botulinum*, *Clostridium* butyricum and *Clostridium* baratii can also produce type E and type F botulinum toxins. Currently, eight serotypes (A-H) of botulinum toxin are known, among which types A,B,and E are the most common causes of human poisoning. Symptoms caused by type A toxin are generally more severe than those caused by type B (Cherington, 1998), while type F poisoning is occasionally reported (Sobel, 2005; Filozov et al., 2012). The primary route of poisoning is foodborne botulism,but injection-induced botulinum toxin poisoning has also been reported (Kılboz et al., 2023; Goerttler et al., 2024). The patient described in this article had injection-induced botulism, consistent with previously reported cases,but the sequelae of right vocal cord paralysis are relatively rare. The incubation period for foodborne botulism is typically from 12 to 72 h, while for injection-induced poisoning, it is generally within 6 days. The length of the incubation period is related to the botulinum toxin serotype (Rossetto et al., 2014): type A poisoning has an incubation period of 0–7 days, type B 0–5 days,and type E 0–2 days. A shorter incubation period often indicates more severe disease. Approximately 98% of patients exhibit at least one symptom of cranial nerve dysfunction (Rao et al., 2017). The neurological symptoms of various botulism types are highly consistent, characteristically presenting as symmetrical, descending, flaccid paralysis that begins in the cranially innervated muscle groups (Heilmann et al., 2024). The classic presentation includes the “4D″ signs: diplopia, dysarthria, dysphonia,and dysphagia. Paralysis descends, affecting the neck muscles, upper limb muscles, respiratory muscles, trunk muscles,and lower limb muscles. Respiratory muscle paralysis is the primary cause of death. In this patient, dyspnea,ptosis, swallowing and masticatory weakness,and choking on water appeared 1 day after injection of an illegal botulinum toxin formulation into the bilateral masseter muscles. The rapid progression of the condition was highly consistent with the toxicological characteristics of injection-induced botulism, indicating that injection-induced botulism carries significant clinical risk.

Unlike foodborne and wound botulism,injection-induced botulism presents with symmetrical, descending,flaccid paralysis, representing a weak area in current clinical understanding. Its uniqueness is reflected in the following three aspects: (1) Short incubation period, with disease onset within hours to 72 h after injection, highly consistent with the kinetics of local toxin diffusion (Bhidayasiri and Truong, 2005). (2) Systemic symptoms and signs of poisoning appear in a top-down order following a specific timeline, independent of the poisoning route. Some cases may lack typical cranial nerve signs but instead present with a prominent focal symptom, which can be easily misdiagnosed as idiopathic vocal cord paralysis or intubation-related injury (Ludlow, 2011). (3) Some rare cases present with prominent focal laryngeal paralysis (Carruthers et al., 2008). The possible mechanism involves botulinum toxin spreading from the injection area (e.g., platysma, masseter, frontalis muscles) along fascial planes to the intrinsic laryngeal muscles, causing chemical denervation of the posterior cricoarytenoid and thyroarytenoid muscles. Clinical manifestations include unilateral vocal cord paralysis (slightly more common on the right side),vocal fatigue, choking on water,and exertional dyspnea (Heilmann et al., 2024). Laryngeal paralysis caused by BTX-A injection has the following characteristics: (a) Short incubation period, with symptoms typically appearing within hours to a few days after injection (usually <72 h), consistent with the temporal kinetics of local BTX-A diffusion (Bhidayasiri and Truong, 2005). (b) Mainly manifests as laryngeal dysfunction such as hoarseness and decreased vocal volume, usually without or with only mild systemic cranial nerve symptoms (e.g., diplopia, ptosis) or descending paralysis of botulism (Ludlow CL.,2011). (c) Often presents as incomplete or complete paralysis of the unilateral vocal cord, reflecting the local diffusion effect of BTX-A on the intrinsic laryngeal muscles in the injected area (Carruthers et al., 2008). Although bilateral vocal cord paralysis is rare,it can lead to acute respiratory distress due to glottic stenosis, requiring urgent evaluation and management.

**FIGURE 5:**
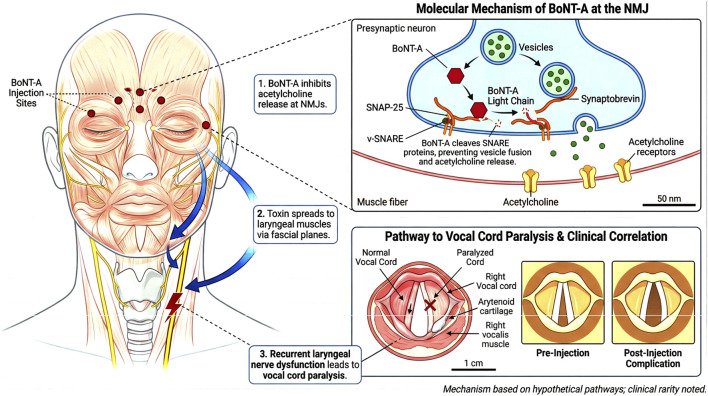
Possible molecular mechanism of Botulinum Toxin Type A-induced unilateral vocal cord paralysis.

Currently, BTX-A is widely used in the field of medical aesthetics (Yi et al., 2022; Rho et al., 2025) (Figures 2A-E). Beauty salons and private studios remain the primary injection sites, accounting for approximately 88.8% of procedures according to research statistics (Xu et al., 2025). Botulinum toxin injection in non-medical facilities cannot guarantee product quality or the operational skill level of injectors, inevitably greatly increasing the risk of botulism for recipients. There is a close relationship between the injected dose of botulinum toxin and its neuromuscular paralytic effect; excessive dosage can lead to a potent and prolonged neuromuscular blockade and even more severe adverse reactions (Hagberg et al., 2024). Its potential complications, especially vocal cord paralysis, although rare, have attracted clinical attention (Heilmann et al., 2024). Symptoms and signs present with a distinct temporal sequence and symmetric descending flaccid paralysis, beginning with cranial nerve involvement. The mechanism primarily involves BoNT blocking the release of acetylcholine at the neuromuscular junction, leading to muscle relaxation (Rossetto et al., 2014). However,its potential side effects, particularly vocal cord paralysis,are rarely reported and have become a clinical concern. The core mechanism of such complications is related to the highly selective toxic effect of BTX-A on the motor nerve terminals of the larynx (Figure 5). As a zinc-dependent endopeptidase, BTX-A specifically cleaves a key protein in the presynaptic membrane of motor neurons—synaptosomal-associated protein of 25 kDa (SNAP-25) (Nemanić et al., 2025; Brin and Burstein, 2023). This action disrupts the structural integrity of the soluble N-ethylmaleimide-sensitive factor attachment protein receptor (SNARE) complex, irreversibly inhibiting the fusion and release of acetylcholine vesicles, ultimately causing chemical denervation of laryngeal muscles (e.g., posterior cricoarytenoid, lateral cricoarytenoid, and thyroarytenoid muscles),manifesting as flaccid paralysis. The patient described in this article developed botulism after receiving injections of an illegal formulation at an unlicensed aesthetic facility and subsequently developed residual right vocal cord paralysis following successful treatment. Although it remains unclear whether this manifestation was caused by botulism or by tracheal intubation, further investigation is required.

Epidemiological data suggest that the incidence of vocal cord paralysis due to botulism is less than 0.1% (Andrew et al., 2015). Patients present with a classic triad—dysphonia, potential respiratory distress, and dysphagia—severely affecting their quality of life. Core diagnostic clues include: (1) Epidemiological history (suspicious food, injection history, drug abuse history); (2) Acute onset, with or without cranial nerve palsy + descending flaccid paralysis; (3) Normal sensation, clear consciousness; (4) Positive botulinum toxin test as the gold standard; (5) Abnormal findings on laryngoscopy and neuromuscular electrophysiological studies. Early diagnosis first requires laryngoscopy, which can reveal structural changes in the epiglottis, vocal cords, etc., but in-depth identification of the lesion requires examinations such as laryngeal electromyography (LEMG) and laryngeal nerve evoked potentials. LEMG is an important tool for diagnosing laryngeal neuromuscular dysfunction. Currently, LEMG is still primarily qualitative and carries significant subjectivity (Lin et al., 2024). For focal paralysis following injection-induced botulism, LEMG may show chronic denervation with early reinnervation changes; evoked potentials are typically preserved, a feature that helps differentiate it from recurrent laryngeal nerve transection injury. Studies have shown that after BTX-A injection poisoning, patient electromyography often exhibits features of myogenic damage, including reduced amplitude and shortened duration of motor unit action potentials, and an increase in polyphasic waves (Zhang et al., 2025). In this patient, LEMG of the right thyroarytenoid and posterior cricoarytenoid muscles showed low amplitude and short duration, consistent with previous research findings.

For the treatment of vocal cord paralysis caused by botulism,in addition to conventional antitoxin therapy, neurotrophic support,and functional rehabilitation training, vocal cord function training and voice therapy should be combined to maximize the recovery of phonatory and swallowing functions. If necessary, local injection of neostigmine into the lesion can be attempted. In this case,the patient showed significant improvement in vocal cord paralysis after receiving local neostigmine injection therapy; however,the specific mechanism and long-term efficacy require further in-depth investigation. Botulism-related laryngeal paralysis results from the potent blockade of laryngeal neuromuscular junctions by BTX-A. When injecting BTX-A in the head and neck region (e.g., platysma, masseter, glabellar, frontal muscles),clinicians should be highly aware of the risk of drug diffusion to the intrinsic laryngeal muscles. They must possess precise anatomical knowledge, employ standardized injection techniques (including accurate control of injection site, depth, and dose), adhere to the principle of the lowest effective dose, and fully inform patients of this rare but serious potential complication.

## 4 Conclusion

This article presents a case of unilateral vocal cord paralysis that persisted following successful treatment for poisoning caused by illegal facial injections of BTX-A. Its pathological essence is focal, chemical, and reversible neuromuscular blockade, rather than permanent nerve transection. Laryngoscopy and laryngeal electromyography are helpful for early diagnosis, and local injection of neostigmine into the affected laryngeal muscles may help alleviate symptoms. As the clinical application of botulinum toxin becomes increasingly widespread, clinicians should remain highly vigilant for laryngeal complications. A full-cycle management system encompassing “safe injection, early identification, and precise rehabilitation” should be established to enhance prevention and control capabilities and improve patient outcomes.

## Data Availability

The original contributions presented in the study are included in the article/supplementary material, further inquiries can be directed to the corresponding author.
